# Exosomal miR-29b of Gut Origin in Patients With Ulcerative Colitis Suppresses Heart Brain-Derived Neurotrophic Factor

**DOI:** 10.3389/fmolb.2022.759689

**Published:** 2022-02-22

**Authors:** Haifeng Lian, Xiaoying S. Zhong, Ying Xiao, Zhe Sun, Yuanyuan Shen, Kaile Zhao, Xingbin Ma, Yanmin Li, Qiong Niu, Max Liu, Don W. Powell, Chengxia Liu, Qingjie Li

**Affiliations:** ^1^ Department of Gastroenterology, Binzhou Medical University Hospital, Binzhou, China; ^2^ Division of Gastroenterology, Department of Internal Medicine, The University of Texas Medical Branch at Galveston, Galveston, TX, United States; ^3^ Department of Gastroenterology, Xiangya Hospital, Central South University, Changsha, China

**Keywords:** inflammatory bowel disease, exosome, microRNA, cardiac remodeling, pro-inflammatory cytokine

## Abstract

**Background and Aims:** While the interplay between heart and gut in inflammatory bowel disease (IBD) has previously been noted, how the inflamed gut impairs heart function remain elusive. We hypothesized that exosomal miRNAs of gut origin induce cardiac remodeling in IBD. Our aim was to identify plasma exosomal miRNAs that not only are of diagnostic value but also contribute to cardiac remodeling in patients with ulcerative colitis (UC).

**Methods:** Plasma exosomes were isolated from UC patients and healthy control subjects and exosomal miRNAs were profiled by next-generation sequencing. Exosomal miR-29b levels in CCD841 CoN colon epithelial cells were detected by RT-qPCR. Exosomes packaged with miR-29b were incubated with H9c2 cells or administered to live mice.

**Results:** The plasma exosomal miRNA profiles of the UC patients were significantly different from that of the controls and 20 miRNAs including miR-29b were differentially expressed. In CCD841 CoN cells, TNFα, IL-1β, and H_2_O_2_ significantly elevated miR-29b in both the cells and their secreted exosomes (*p* < 0.01), suggesting that intestinal epithelium secrets exosomes rich in miR-29b in IBD. In H9c2 myoblast cells, miR-29b modulated multiple genes including brain-derived neurotrophic factor (BDNF). Epithelial cell-derived exosomes packaged with miR-29b also attenuated BDNF and increased cleaved caspase 3, suggestive of apoptosis. Furthermore, tail vein injection of engineered exosomes with high levels of miR-29b suppressed BDNF and augmented cleaved caspase 3 in the heart of adult mouse (*p* < 0.01).

**Conclusion:** Plasma exosomal miRNA profile could be a novel diagnostic approach for IBD. Excessive plasma exosomal miR-29b suppresses critical proteins like BDNF in IBD, leading to cardiac impairment.

## Introduction

Inflammatory bowel diseases (IBD), including ulcerative colitis (UC) and Crohn’s disease (CD), are idiopathic, chronic and relapsing inflammatory conditions of the gastrointestinal tract, which usually are characterized by diarrhea, rectal bleeding, abdominal pain, fatigue, and weight loss. IBD can be debilitating and sometimes leads to life-threatening complications. Up to 50% of IBD patients experience extraintestinal manifestations, commonly in the joints, skin, bones, eyes, kidneys, and liver (Vavricka et al., 2015). Accumulating evidence also suggests that IBD is associated with increased risk of hospitalization for heart failure (Kappelman et al., 2011; Kristensen et al., 2014), developing ischemic stroke (Huang et al., 2014), and myocardial infarction (Choi et al., 2019; Panhwar et al., 2019). However, the organ-to-organ communication between gut and heart has been insufficiently investigated and the molecular mechanisms by which IBD induces cardiac disorders remain largely unknown.

Exosomes are small extracellular vesicles contributing to cell-to-cell communication and their pathophysiological relevance and therapeutic potential are emerging. Particularly, exosomes are thought to play an important role in inter-organ communication under normal and pathological conditions by transferring proteins, DNA, mRNA, and microRNA (miRNA) stably in various biofluids (Kita et al., 2019; Verweij et al., 2019). It is well known also that sustained chronic inflammation elevates pro-inflammatory cytokines and reactive oxygen species in the gut of IBD patients (Strus et al., 2009; Friedrich et al., 2019). Another hallmark of IBD is dysbiosis, characterized by depleted salutary bacteria such as *B. adolescentis* and *F. prausnitzii*, and proliferation of virulent bacteria such as *Proteobacteria* (Chu et al., 2018; Ma et al., 2018; Moustafa et al., 2018). Of note, the dysbiosis does not dissipate even after IBD patients enter remission. Pro-inflammatory cytokines, reactive oxygen species, and bacterial pathogens can modulate host miRNA expression and exosome excretion (Maudet et al., 2014), suggesting that exosomes of gut origin may contribute to extraintestinal manifestations of IBD such as cardiac disorders. While circulating miRNAs in patients with IBD have been investigated (Paraskevi et al., 2012; Schönauen et al., 2018), exosomal miRNA profiles in the peripheral blood of UC patients have not been fully characterized. We hypothesized that IBD dysregulates exosomal miRNAs in the peripheral blood, leading to adverse cardiac remodeling.

Brain-derived neurotrophic factor (BDNF) is a key player in cardiovascular system (Pius-Sadowska and Machaliński, 2017). Recent studies show that constitutive myocardial BDNF/TrkB signaling is required for normal cardiac contraction and relaxation. Cardiac-specific conditional TrkB knockout mice display accelerated heart failure progression under pressure overload (Feng et al., 2015). In patients with UC, BDNF levels have been found to be significantly lower in the plasma and nerve structures, compared to the healthy control subjects (Johansson et al., 2007; Johansson et al., 2008). In contrast, treatment of rat colon smooth muscle tissue with TNF-α and IL-1β resulted in a significant increase in the protein expression of BDNF, suggesting that colitis elevates BDNF levels inside the colon wall (Al Qudah et al., 2020). Yet, whether and how cardiac BDNF is repressed by colitis remain to be determined. We postulated that exosomal miRNAs, such as miR-29b, might suppress BDNF in the adult heart. It has been reported that miR-29b levels are elevated in the inflamed colonic mucosa of IBD patients (Fasseu et al., 2010). High levels of miR-29b were also detected in the plasma of IL10^−/−^ mice (Viennois et al., 2017). A recent study demonstrated that circular homeodomain-interacting protein kinase 3 improves repair of the intestinal epithelium by reducing miR-29b availability (Xiao et al., 2021).

In the present study, we isolated exosomes from the plasma of UC patients and healthy control subjects and performed miRNA profiling using next-generation sequencing. We identified 13 miRNAs, including miR-29b, that were significantly upregulated and 7 miRNAs that were significantly decreased in patients with UC compared to control subjects. We found that pro-inflammatory cytokines tumor necrosis factor alpha (TNFα) and interleukin-1 beta (IL-1β), and hydrogen peroxide elevated miR-29b levels in intestinal epithelial cells. In H9c2 myoblast cells, overexpression of miR-29b modulated multiple genes including BDNF (Feng et al., 2015). Furthermore, engineered exosomes with high levels of miR-29b suppressed BDNF and increased Caspase 3 cleavage both in H9c2 cells and in the mouse heart, demonstrating that exosomal miR-29b induces molecular remodeling in the adult heart.

## Results

### Patient Information

Age, sex and clinical characteristics of 6 patients with UC are shown in Supplementary Table S1. Ages of the 4 control subjects (2 males and 2 females) are between 50 and 60 and with an average of 54. All control subjects had smooth and normal-looking colonic mucosa under a colonoscope.

### Overview of miRNA Transcriptome in Plasma Exosomes

**FIGURE 1:**
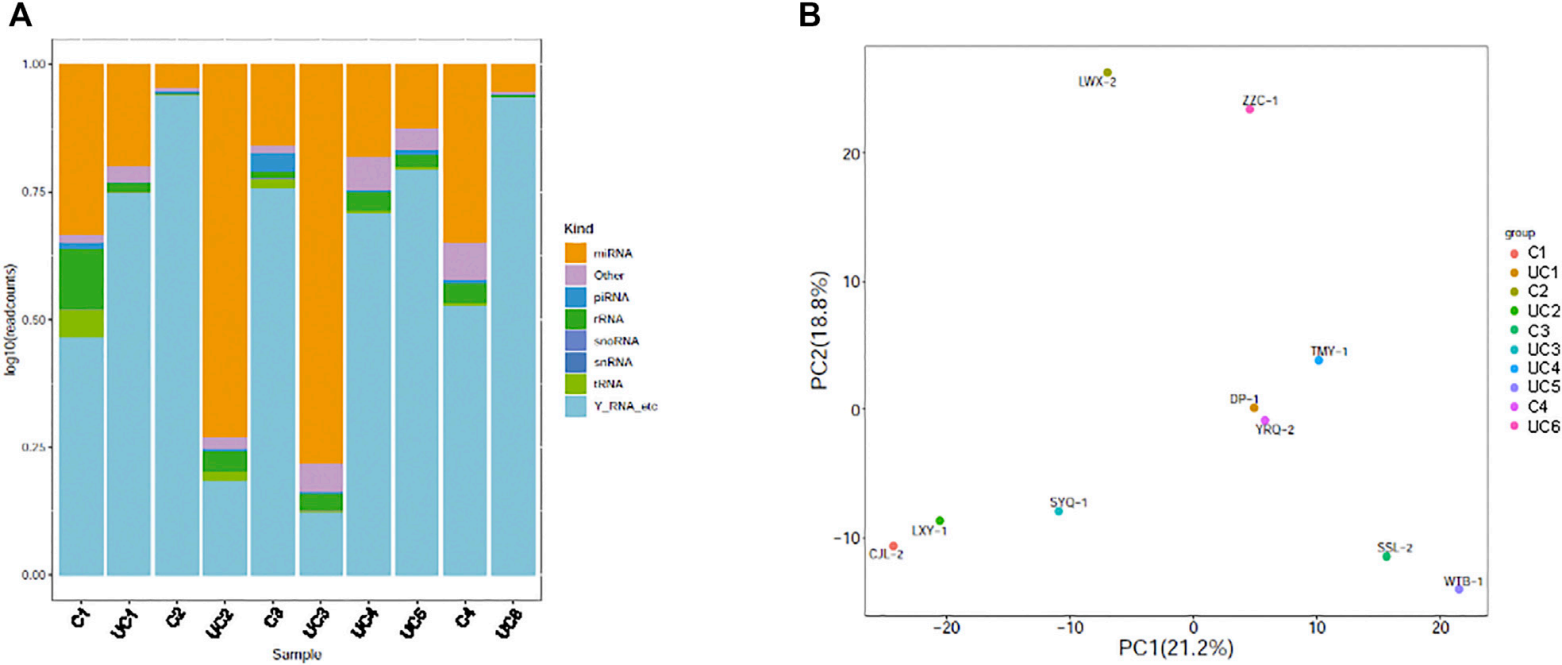
Genome-wide survey of human small RNAs in plasma exosomes. **(A)** Stacked bar charts showing relative distribution of sequencing reads corresponding to 8 types of non-coding RNAs. **(B)** Principal component analysis (PCA) plot showing the first two principal components using normalized read counts. Each dot indicates a sample. C = control; UC = ulcerative colitis.

Small RNA sequencing of the 10 samples revealed that plasma exosomes contain multiple types of small RNAs, with miRNA as the primary small RNA in two of the samples (Figure 1A). Principal component analysis (PCA) was performed, and the scatter plot with two principal components (PC1 21.2%, PC2 18.8%) showed that all the samples spread out with neither the patient samples nor the control samples grouped together (Figure 1B), indicating high variations between the samples.

On average for both the UC patients and the control subjects, we obtained 12.5 million total reads and 12 million clean reads per sample, of which 11.7 million reads aligned to the reference human genome sequence (97.24%, Table 1). The number of expressed miRNAs ranged from 751 to 1,149 per sample, with an average of 939 known miRNAs. The top 10 highly expressed miRNAs in the plasma exosomes were miR-151a-3p, miR-126-3p, miR-451a, miR-146a-5p, miR-26a-5p, let-7i-5p, miR-30d-5p, miR-24-3p, let-7f-5p, and miR-140-3p.

**TABLE 1:** Read counts and known miRNA numbers detected in all 10 samples. Average of each column.

Sample name	Total reads	Clean reads	Mapping reads	Mapping%	No. of miRNA
C1	12,326,850	12,110,868	11,950,445	98.68	925
UC1	12,373,525	12,108,003	11,808,595	97.53	927
C2	11,418,530	10,611,621	10,164,078	95.78	751
UC2	12,378,300	12,110,986	11,989,411	99.00	1,077
C3	12,428,550	12,111,897	11,951,939	98.68	840
UC3	12,835,325	12,350,972	12,042,209	97.50	1,149
UC4	12,819,575	12,196,113	11,686,569	95.82	1,037
UC5	12,947,325	12,038,548	11,550,205	95.94	832
C4	12,971,950	12,211,102	11,783,293	96.50	1,054
UC6	12,841,325	12,119,499	11,746,403	96.92	804
Average	**12,534,126**	**11,996,961**	**11,667,315**	**97.24**	**939.2**

Overall, miRNAs are more abundant in the exosomes of UC patients compared to that of control subjects (*p* < 0.01). Among the total reads, 56.15% were unique in the patients with UC, 27.76% were unique in the control subjects, and only 16.09% were common between both groups (online Supplementary Figure S1). The small RNA sequencing data has been deposited in the NCBI SRA database (Accession No. PRJNA742845. URL: https://www.ncbi.nlm.nih.gov/sra/PRJNA742845).

We were able to discover 456 new miRNAs after aligning our sequencing data to the reference human genome. The top 6 new miRNAs with read count >200 and total score >100 are listed in Supplementary Table S2. The most promising new miRNA (ID: chr20_10,902) is located at chromosome 20 and has a sequence of ucc ggg aug ggc acu cug cuc for its mature miRNA, which we named miR-10902. It had 19,423 clean reads and a total score of 9,903.8 (online Supplementary Figure S2).

### Identification of Differentially Expressed Exosomal miRNAs in Patients With UC

**FIGURE 2:**
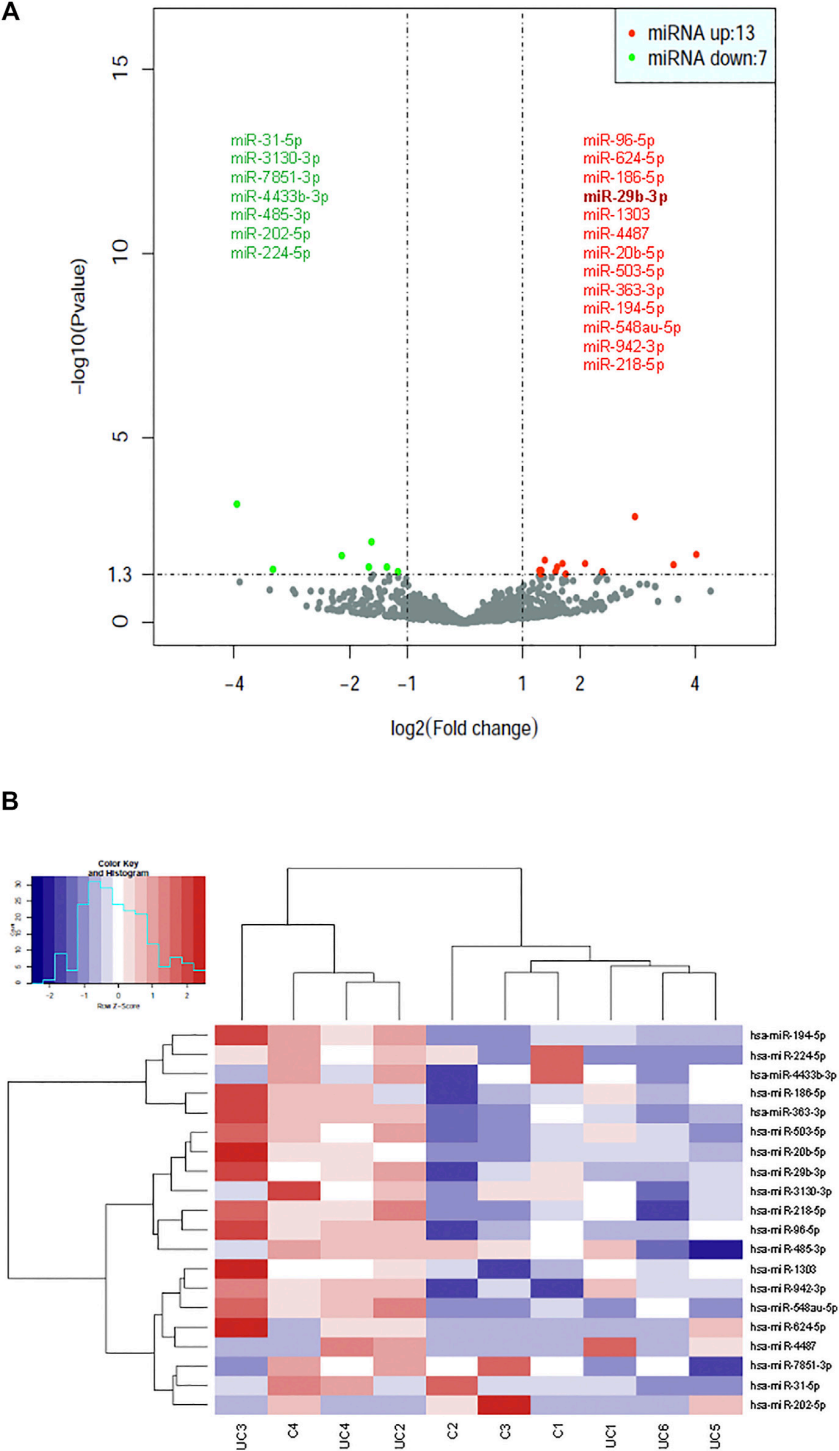
Identification of dysregulated exosomal miRNAs in patients with UC by statistical analysis of small RNA-seq data. **(A)** Volcano plot representation of differential expression analysis of miRNAs in patients with UC vs healthy control subjects. The miRNAs are colored if they pass the thresholds for *p* value and log fold change, red if they are upregulated and green if they are downregulated. **(B)** Hierarchical clustering of normalized exosomal miRNA levels for the 20 miRNAs having statistical significance for differential expression between UC patients and control subjects.

Statistical analysis of normalized miRNA sequencing data identified 20 differentially expressed miRNAs (Bonferroni adjusted *p*-value < 0.01), 13 of which (miR-29b-3p, 96-5p, 624-5p, 186-5p, 1,303, 4,487, 20b-5p, 503-5p, 363-3p, 194-5p, 548au-5p, 942-3p, and 218-5p) were upregulated and 7 (miR-31-5p, 3130-3p, 7851-3p, 4433b-3p, 485-3p, 202-5p, and 224-5p) downregulated in patients with UC (Figure 2A). A hierarchical cluster analysis using Spearman’s correlation distance was performed for all 20 deregulated miRNAs (Figure 2B).

### Prediction of miRNA Target Genes and Gene Ontology Analysis

Target genes of each miRNA were predicted using miRDB, miRWalk, TargetScan, and miRTarBase for all 20 miRNAs that were either upregulated or downregulated in UC patients. Based on the data sets, gene interaction networks were constructed for either the 13 upregulated miRNAs (online Supplementary Figure S3) or the 7 downregulated miRNAs (online Supplementary Figure S4). Venn diagrams illustrating the intersections among the four prediction data sets were created for all 20 differentially expressed miRNAs in patients with UC (online Supplementary Figure S5).

**FIGURE 3:**
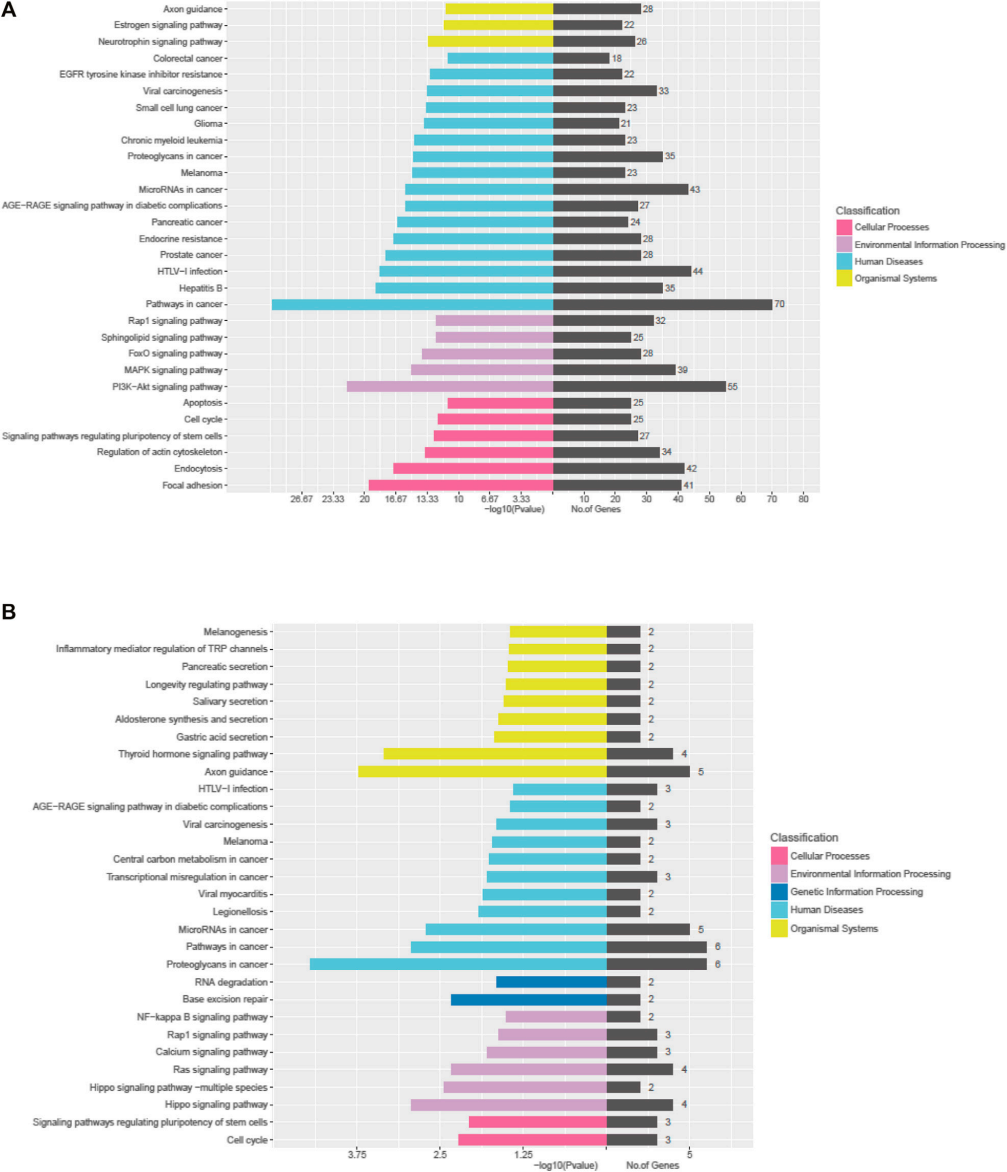
KEGG pathway analyses of the dysregulated exosomal miRNAs. **(A)** KEGG pathway analysis of upregulated miRNAs. **(B)** KEGG pathway analysis of downregulated miRNAs.

**FIGURE 4:**
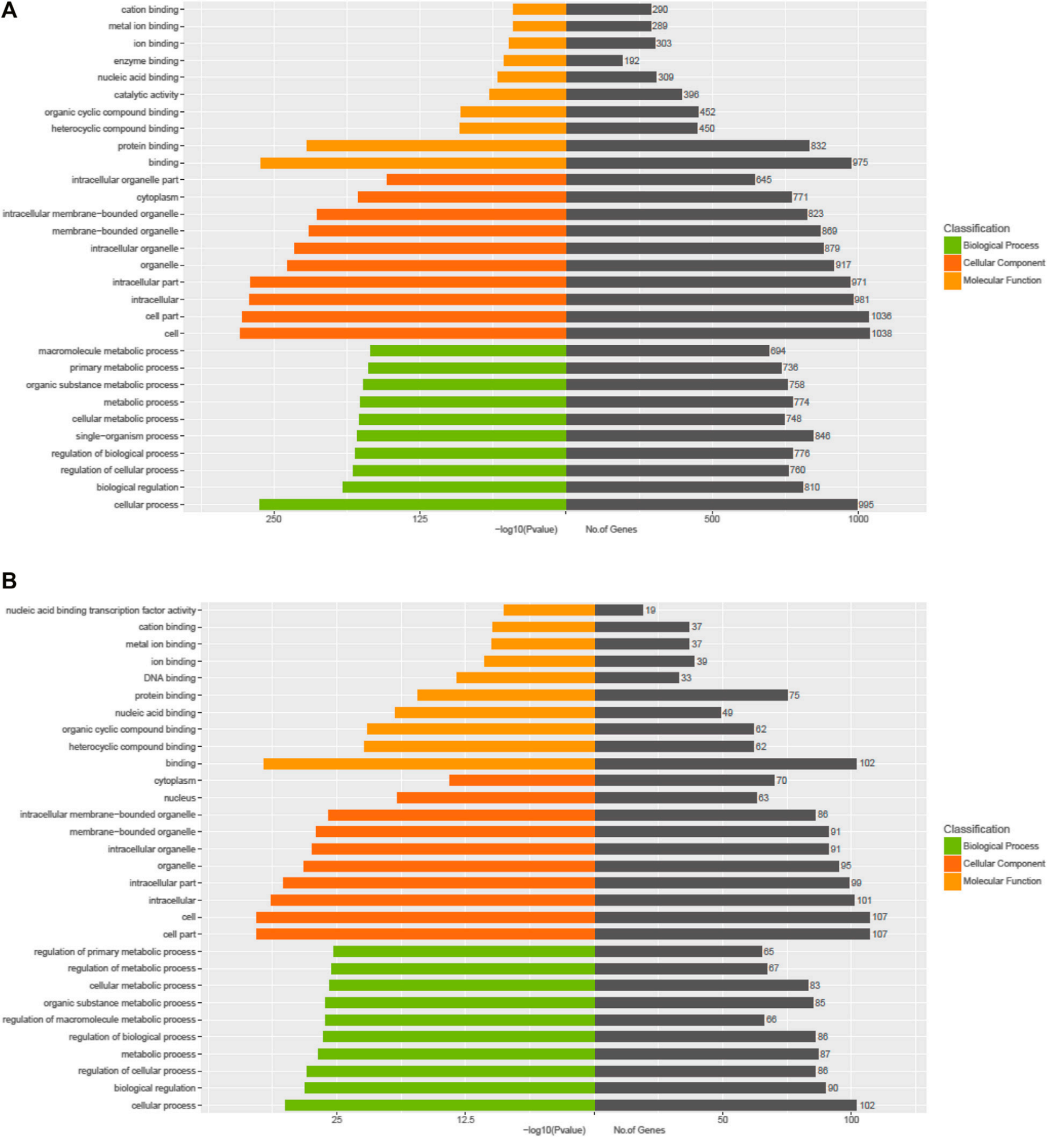
GO enrichment analyses of the exosomal miRNAs dysregulated in UC patients compared to the control subjects. **(A)** Upregulated miRNAs. **(B)** Downregulated miRNAs.

The Kyoto Encyclopedia of Genes and Genomes (KEGG) pathway analysis for the target genes of dysregulated miRNAs revealed that the upregulated miRNAs were highly associated with multiple signaling pathways, including neurotrophin signaling pathway, FoxO signaling pathway, apoptosis, and regulation of actin cytoskeleton (Figure 3A, online Supplementary Figure S6), whereas the downregulated miRNAs were enriched in pathways such as inflammatory mediator regulation of TRP channels, RNA degradation, NF-kappa B signaling pathway, and calcium signaling pathway (Figure 3B, online Supplementary Figure S7), which are critical to cardiac homeostasis. For further analyses of the target genes, we performed GO enrichment analyses for the upregulated miRNAs (Figure 4A) or downregulated miRNAs (Figure 4B). We obtained the GO terms annotating the target genes from the GO database, and then performed the enrichment analysis for each GO term by a conditional hypergeometric test. Gene function annotations were classified into three categories: biological process, cellular component, and molecular function. The top ten enriched GO terms of each category according to the enrichment scores of the GO terms are presented in Figure 4.

### TNFα, IL-1β, and H_2_O_2_ Elevate miR-29b Levels in Human Colon Epithelial Cells and Their Exosomes

**FIGURE 5:**
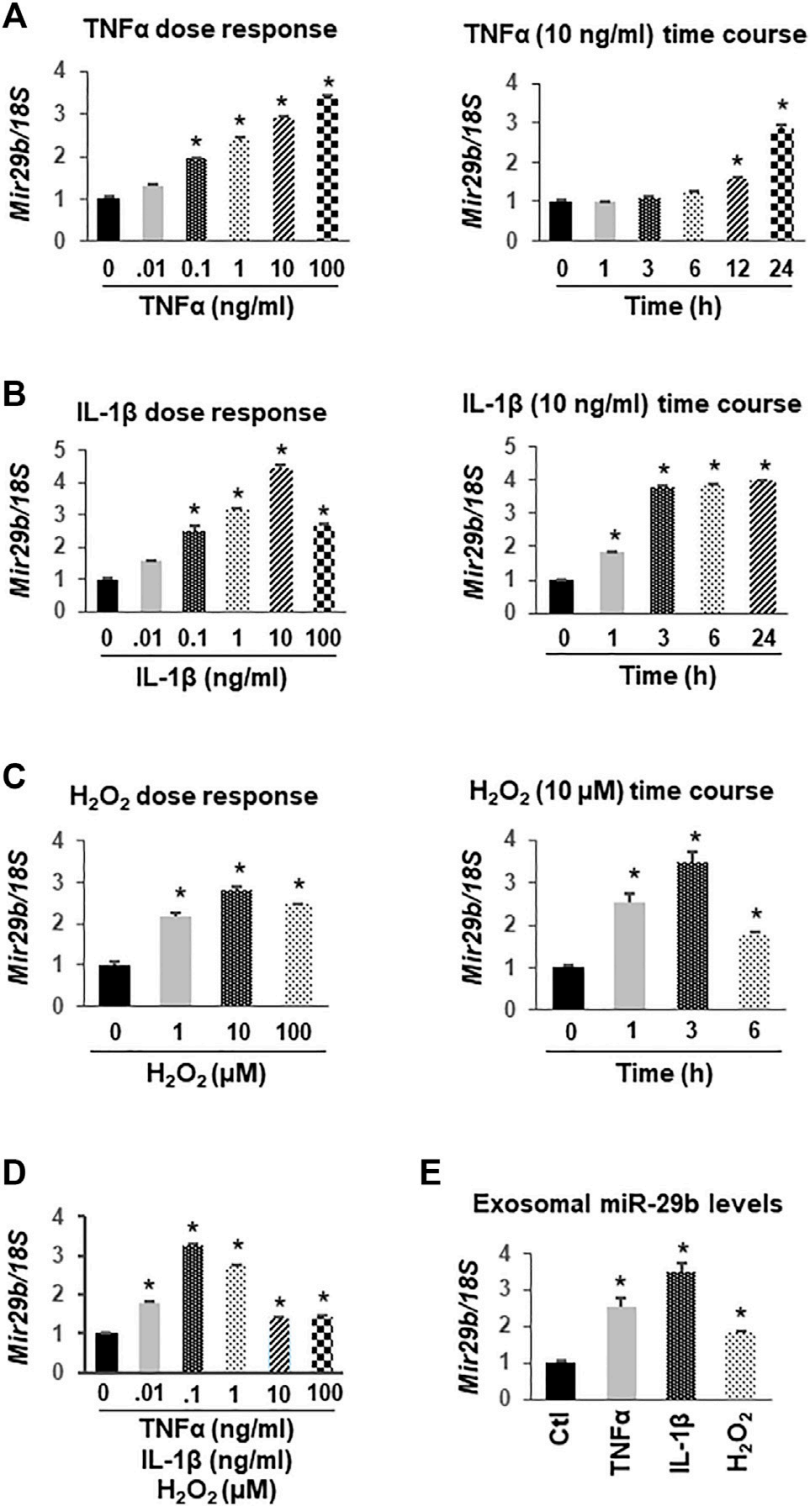
Upregulation of miR-29b gene (*Mir29b*) expression by TNFα, IL-1β, and H_2_O_2_ in CCD841 CoN colon epithelial cells. CCD841 CoN cells were incubated with serial doses of TNFα, IL-1β, H_2_O_2_ or their combination for 24 h, or with 10 ng/ml TNFα, 10 ng/ml IL-1β, or 10 µM of H_2_O_2_ for various times as indicated. *Mir29b* gene expression was quantitated by TaqMan-based RT-qPCR. **(A)** TNFα dose- and time-dependently elevated *Mir29b* levels. **(B)** IL-1β increased *Mir29b* in a dose- and time-dependent manner. **(C)** H_2_O_2_ dose- and time-dependently up-regulated *Mir29b*. **(D)** Combination of TNFα, IL-1β, and H_2_O_2_ augmented *Mir29b* expression. **(E)** Exosomal levels of *Mir29b* were elevated by 10 ng/ml TNFα, 10 ng/ml IL-1β, or 10 µM H_2_O_2_ after 24 h incubation. n = 3 independent experiments. *p < 0.01 *vs.* control (concentration or time zero).

Based on the literature and our preliminary work showing a potential role of miR-29b in the gut-heart communication, we selected miR-29b for further molecular studies. To determine if colon epithelial cells overexpress miR-29b under inflammatory conditions, we incubated CCD841 CoN colon epithelial cells with TNFα, IL-1β, H_2_O_2_, or their combination. RT-qPCR analyses showed that TNFα upregulated miR-29b in a dose-dependent (Figure 5A *,* left panel) and time-dependent (Figure 5A *,* right panel) manner. Even a low dose (0.1 ng/ml) of TNFα significantly increased miR-29b levels (Figure 5A, *p* < 0.01), which is physiologically relevant because the serum levels of TNFα in patients with UC range from 0.1 to 0.5 ng/ml (Maeda et al., 1992). IL-1β also elevated miR-29b in a dose-dependent manner, with 10 ng/ml being most effective (Figure 5B, left panel). A higher dose of IL-1β (100 ng/ml) still significantly increased miR-29b, but was not as effective as 10 ng/ml. The lowest effective dose of IL-1β is 0.1 ng/ml, which is still much greater than the IL-1β concentration in most patients with UC (1–5 pg/ml) (Vounotrypidis et al., 2013). Ten ng/ml of IL-1β significantly upregulated miR-29b in just 1 hour, and the expression level reached a plateau in 3 h (Figure 5B, right panel. *p* < 0.01). The increases in miR-29b expression was sustained from 3 to 24 h. In comparison, 10 ng/ml TNFα took 12 h to show a significant effect (Figure 5A). H_2_O_2_ also concentration-dependently elevated miR-29b with 10 µM being the most potent (Figure 5C, left panel. *p* < 0.01). The changes in miR-29b expression were sustained from hour 1 to 6 when exposed to a fixed dose of H_2_O_2_ (10 μM, Figure 5C *,* right panel. *p* < 0.01), which is also physiologically relevant since the H_2_O_2_ levels in patients with UC have a wide range and could be up to 500 µM (Cao et al., 2004). While either TNFα, IL-1β, or H_2_O_2_ at 0.01 ng/ml (µM for H_2_O_2_) failed to elevate miR-29b, their combination significantly upregulated miR-29b (Figure 5D), suggesting an additive effect. All higher concentrations of TNFα, IL-1β, and H_2_O_2_ in combination significantly increased miR-29b.

To demonstrate that miR-29b levels were concurrently upregulated in exosomes, we collected medium from the CCD841 CoN cells treated with 10 ng/ml TNFα, 10 ng/ml IL-1β, or 10 µM H_2_O_2_ for 24 h, isolated exosomes, and assessed miR-29b levels using TaqMan-based RT-qPCR. The expression levels of miR-29b in the exosomes were significantly increased by TNFα, IL-1β, or H_2_O_2_ (Figure 5E), suggesting colon epithelial cells as a major origin of exosomal miR-29b under inflammatory conditions such as IBD.

### miR-29b Alters the Expression Levels of Multiple Genes in H9c2 Cells and Exosomal miR-29b Suppresses BDNF *in vitro* and *in vivo*

**FIGURE 6:**
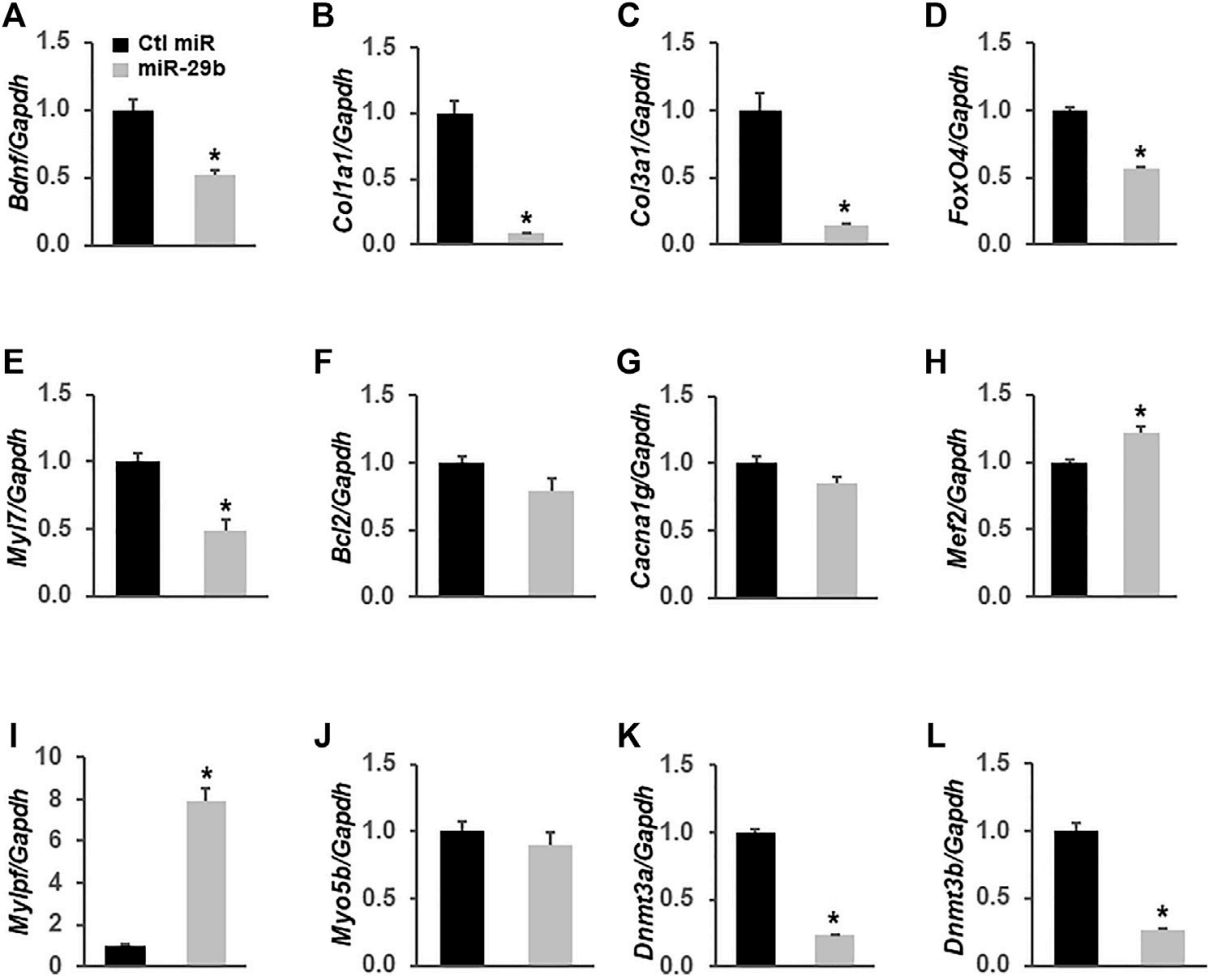
miR-29b modulates the expression levels of multiple genes related to heart function in H9c2 cells. miR-29b or negative control miRNA was overexpressed in H9c2 cells by transient transfection. TaqMan-based RT-qPCR was performed to determine the expression levels of *Bdnf* **(A)**, *Col1a1* **(B)**, *Col3a1* **(C)**, *Fox O 4* **(D)**, *Myl7* **(E)**, *Bcl2* **(F)**, *Cacna1g* **(G)**, *Mcf2* **(H)**, *Mylpf* **(I)**, *Myo5b* **(J)**, *Dnmt3a* **(K)**, and *Dnmt3b* **(L)**. *Gapdh* served as an internal control. n = 3 independent experiments. *p < 0.01 *vs.* negative control miRNA (Ctl miR).

To demonstrate that miR-29b induces molecular remodeling in cardiomyocytes, we first overexpressed miR-29b in H9c2 rat myoblast cells and assessed the expression levels of multiple genes related to heart function and diseases via RT-qPCR. We found that forced expression of miR-29b significantly suppressed *Bdnf* (Figure 6A), *Col1a1* (Figure 6B), *Col3a1* (Figure 6C), *Fox O 4* (Figure 6D), *Myl7* (Figure 6E), *Dnmt3a* (Figure 6K), and *Dnmt3b* (Figure 6L). In addition, *Mef2* (Figure 6H) and *Mylpf* (Figure 6I) were significantly upregulated by miR-29b. We also found that the expression levels of *Bcl2* (Figure 6F), *Cacna1g* (Figure 6G), and *Myo5b* (Figure 6J) remained unchanged in response to forced expression of miR-29b. These findings suggest that miR-29b overexpression stimulates cardiac remodeling and may adversely affect heart function.

**FIGURE 7:**
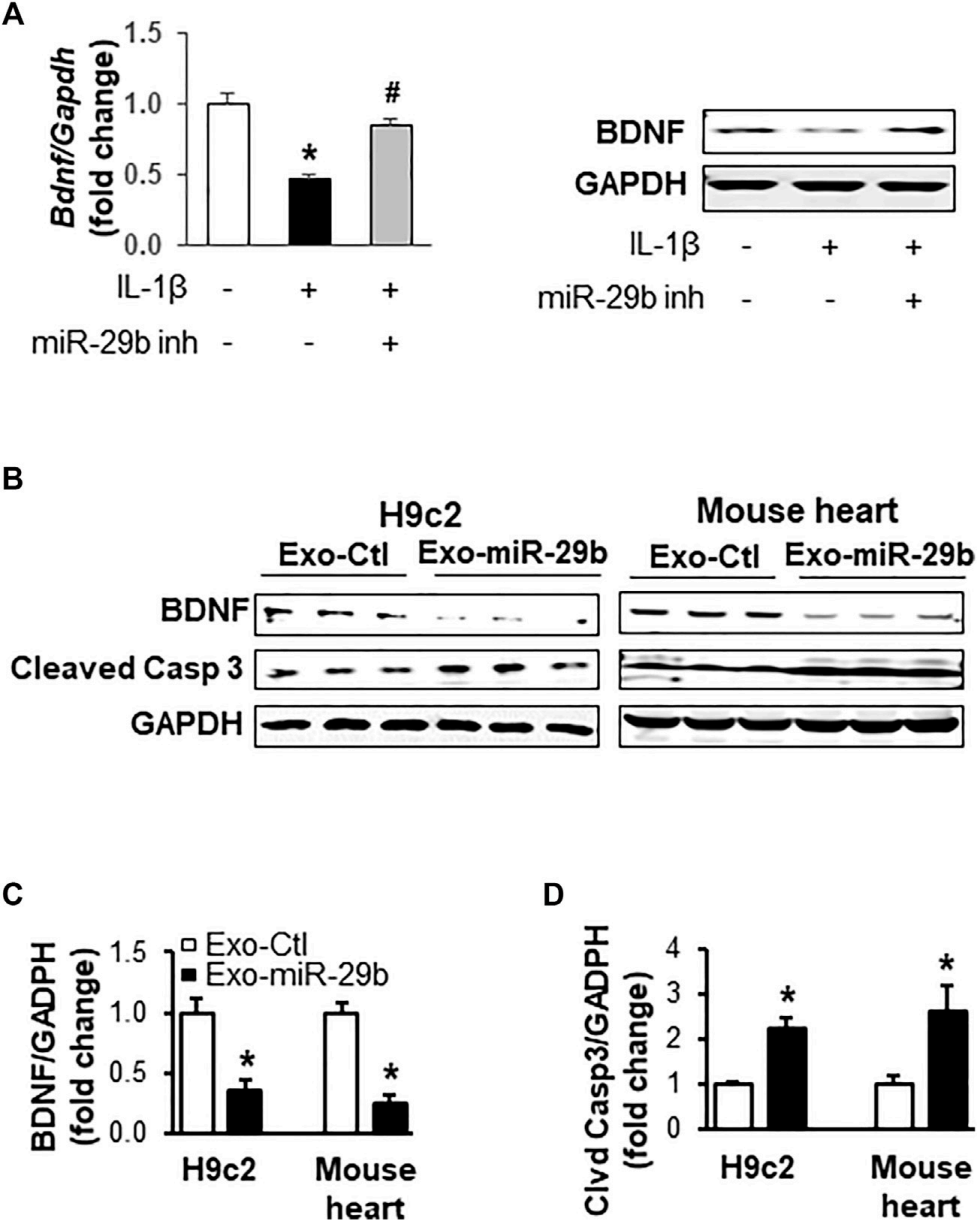
Exosomes rich in miR-29b suppress BDNF and enhance cleaved caspase 3 *in vitro* (cell culture) and *in vivo* (mouse tail vein injection). **(A)** The medium from IL-1β-treated CCD841 CoN cells suppressed BDNF mRNA (left panel) and protein levels (right panel) in H9c2 cells. CCD841 CoN cells were transfected with miR-29b inhibitor (inh) and treated with 10 ng/ml IL-1β. The medium without IL-1β was collected and used to treat H9c2 cells for 24 h. **(B)** Representative images of Western blots showing exosomal miR-29b decreased BDNF *in vitro* and *in vivo*. Bar graphs show the relative band densities of BDNF **(C)** and cleaved caspase 3 **(D)** after normalization to the housekeeping gene GAPDH. *n* = 3 independent experiments for H9c2 cells. *n* = 5 for *in vivo* experiments. *p < 0.01 vs. controls. CCD841 CoN-derived or mouse plasma exosomes were transfected with miR-29b or control miRNA. The CCD841 CoN-derived exosomes loaded with rno-miR-29b-3p mimics were added to H9c2 cells and incubated for 24 h, followed by protein isolation and Western blot analysis. Adult mice were given the mouse exosomes transfected with mmu**-**miR-29b-3p mimics or control miRNA through tail vein injection, and the animals were euthanized 24 h later. Individual hearts were collected for Western blot analysis of proteins.

To investigate if miR-29b derived from CCD841 CoN cells represses BDNF, an important growth factor that helps maintain cardiac homeostasis (Fulgenzi et al., 2015; Kermani and Hempstead, 2019) and directs the response of the cardiovascular system to acute and chronic injury (Kermani and Hempstead, 2019), in H9c2 cells, we transfected CCD841 CoN cells with miRNA control or miR-29b inhibitors and treated the cells with 10 ng/ml of IL-1β for 24 h before switching to fresh medium without IL-1β. Incubation of H9c2 cells with the culture medium of CCD841 CoN cells showed that IL-1β treatment of CCD841 CoN cells led to a suppression of BDNF mRNA (Figure 7A, left panel) and protein (Figure 7B, right panel); the suppression was mitigated by miR-29b inhibitors, suggesting that CCD841 CoN-secreted miR-29b mediates, at least partially, the reduction of BDNF.

We further investigated if miR-29b packaged in exosomes suppresses BDNF *in vitro* and *in vivo*. Western blot analyses revealed that CCD841-derived exosomes with high levels of miR-29b significantly downregulated BDNF protein levels in H9c2 cells, compared to those treated with exosomes containing negative control miRNAs (Figure 7B *,* left panel and Figure 7C, *p* < 0.01). Concurrently, cleaved Caspase 3 was significantly enhanced (Figure 7B left panel and Figure 7D, *p* < 0.01), indicative of apoptosis. More importantly, tail vein injection of mouse plasma exosomes transfected with miR-29b led to a significant decrease in BDNF protein (Figure 7B right panel and Figure 7C, *p* < 0.01) and a concomitant increase of cleaved Caspase 3 (Figure 7B right panel and Figure 7D, *p* < 0.01) in the heart of adult mice, which were in strong agreement with the *in vitro* data obtained from the H9c2 cells.

## Discussion

Circulating exosomes maintain interactions between the inflamed gut and other organs in patients with IBD, and may contribute to the extraintestinal manifestations of IBD, including cardiac remodeling. However, the cargo in plasma-derived exosomes of patients with UC has not been fully characterized and their roles in gut-heart crosstalk remain unclear. Profiling of exosomal miRNA and identification of specific miRNAs that induce molecular remodeling in the adult heart will help elucidate the mechanisms through which inflamed gut conveys disease signals to distal organs in the body. This knowledge might facilitate the development of novel therapies for IBD-induced heart diseases and other extraintestinal manifestations of IBD. In this study, we isolated plasma exosomes from patients with UC and control subjects and profiled miRNAs using next generation sequencing. When compared to controls, we identified 13 miRNAs, including miR-29b, as being significantly upregulated and 7 miRNAs downregulated in the plasma exosomes of UC patients. We characterized the role of exosomal miR-29b in cardiac remodeling in H9c2 cells and in the adult mouse heart. We found that colonic epithelial cells over-express miR-29b, not only within the cells but also inside their secreted exosomes, in response to TNFα, IL-1β, and H_2_O_2_, which are all increased in patients with IBD (Strus et al., 2009; Friedrich et al., 2019). We also found that forced expression of miR-29b altered multiple proteins including BDNF in H9c2 cells. More importantly, engineered exosomes containing miR-29b suppressed BDNF in both H9c2 cells and live animals, providing a mechanistic insight into cardiac impairment in IBD.

IBD is difficult to diagnose due to nonspecific and variable symptoms, and lack of reliable diagnostic markers and tests. miRNAs are emerging as potential markers for many diseases. Many studies on miRNAs in IBD have focused on single miRNA and its association with a target gene or group of genes. Recently, Viennois et al. identified a serum miRNA signature including miR-29b that may distinguish individuals at risk and might be used to evaluate the response to therapeutics in mouse models (Viennois et al., 2017). However, those animal models do not recapitulate most of the common extraintestinal manifestations of human IBD. Our data showed that UC patients have exosomal miRNA profiles quite distinct from those of control subjects. Specifically, plasma exosomes in UC patients contained significantly more abundant miRNAs; more than 56% of miRNAs were unique in patients with UC whereas only 28% in healthy controls; and 20 miRNAs were differentially expressed. Thus, plasma exosomal miRNA profiles could be used for diagnosis of UC or evaluation of therapeutic responses.

Among the 20 differentially expressed exosomal miRNAs in our study, miR-29b and miR-194 were found to be significantly increased in the colonic mucosa of both CD and UC patients; miR-20b appeared to be upregulated in the mucosal tissue and peripheral blood of UC patients; and miR-548 was elevated in the colonic mucosa of UC but decreased in CD patients (Fisher and Lin, 2015). In fact, miR-29b has been found to be commonly upregulated both in quiescent and in inflamed colonic mucosa of IBD patients (Fasseu et al., 2010). Overexpression of miR-29b was also found in the serum of IL10^−/−^ mice (Viennois et al., 2017). More importantly, there is evidence that miR-29b contributes to development (Yang et al., 2020), molecular remodeling (Yang et al., 2020), fibrosis (van Rooij et al., 2008), aging (Lyu et al., 2018), and muscle atrophy (Li et al., 2017) of the heart, demonstrating its necessity in the cardiac system. In CCD841 CoN cells, we found that miR-29b levels were significantly upregulated by TNFα, IL-1β, H_2_O_2_, or their combination in a concentration- or time-dependent manner. The exosomes secreted by CCD841 CoN cells also had significantly higher levels of miR-29b after stimulation by TNFα, IL-1β or H_2_O_2_. These findings suggest that high levels of proinflammatory cytokines and reactive oxygen species in the inflamed intestine of IBD stimulate the intestinal epithelial cells to secret exosomes rich in miR-29b, although we cannot rule out the possibility that other factors such as dysbiotic gut microbiota may dysregulate miR-29b as well. We identified a group of genes, including BDNF, that were significantly altered by miR-29b overexpression in H9c2 cells. Exosomes loaded with miR-29b also significantly suppressed BDNF both in H9c2 cells and in the heart of adult mouse, providing direct evidence that the engineered exosomes were able to deliver miR-29b not only in cultured cells but also in live animals. This supports our notion that exosomes originated in the inflamed colon of IBD can transport disease signal from the intestine to the heart and likely other organs whereby molecular remodeling occurs, which could be detrimental or pathogenic. Recent studies suggest that disturbances/fluctuations in BDNF synthesis and function are associated with devastating CVDs such as heart failure, arrhythmia, and MI, indicating its importance to the cardiac system (Kadowaki et al., 2016; Pius-Sadowska and Machaliński, 2017; Panhwar et al., 2019). BDNF preserves cell viability and function in the brain (L. Neto et al., 2011) and is essential for heart development (Pius-Sadowska and Machaliński, 2017). In adult mammals, BDNF controls autonomic transmission to the heart and exerts prominent angiogenic effects (Feng et al., 2015). Notably, tropomyosin-related kinase receptor B (TrkB), the BDNF receptor, is present in the myocardium (Okada et al., 2012). Several studies found that BDNF/TrkB signaling is required for the heart to fully contract and relax (Feng et al., 2015; Fulgenzi et al., 2015). Our data suggest that chronic colitis impedes heart function, at least partially, by elevating exosomal miR-29b to disrupt BDNF signaling and that BDNF agonists might offer therapeutic and preventive benefit to the patients with IBD.

A limitation of this study is that the patient and control subject numbers were relatively small. In the future, a full miRNome profiling of plasma exosomes from a larger number of IBD patients with diverse ethnicities and patients with irritable bowel syndrome (IBS) will be needed to establish a panel that shows the greatest diagnostic value in the clinic, particularly distinguishing IBD from IBS patients with a simple blood draw rather than an invasive colonoscopy. Delivery of miR-29b inhibitors in exosomes should be investigated in different models of colitis to evaluate its therapeutic potential for IBD and some of its extraintestinal manifestations. Future studies should also focus on the therapeutic and preventive potential of BDNF agonists for the cardiac disorders associated with IBD and other inflammatory diseases.

This study serves as a proof of principle that exosomal miRNA profiles could be an innovative diagnostic approach and that dysregulated exosomal miRNAs contribute to extraintestinal manifestations of IBD. Our findings suggest that over-expressed miR-29b by colitis in the intestine can be packaged in exosomes, which are subsequently released into the blood stream. The plasma exosomes of gut origin can be taken up by cardiomyocytes where they release the cargo causing molecular remodeling by dysregulating critical signaling molecules like BDNF, leading to heart diseases eventually.

## Methods

### Reagents

Human recombinant TNFα (Cat. #Z01001) and IL-1β (Cat. #Z02922) were purchased from GenScript (Piscataway, NJ) and H_2_O_2_ from Sigma (St. Louis, MO). Rno-miR-29b-3p mimics (MBS8302610), mmu-miR-29b-3p mimics (MBS8300641), hsa-miR-29b-3p inhibitor (MBS8292839), and negative control miRNAs were purchased from MyBioSource (San Diego, CA).

### Cell Culture

CCD841 CoN (Cat. # CRL-1790) and H9c2 (Cat. # CRL-1446) cells purchased from American type culture collection (ATCC, Manassas, VA) were maintained in DMEM medium supplemented with 10% FBS and 0.1% penicillin-streptomycin solution (Kline et al., 2019). Transient transfection of miRNA mimics in H9c2 cells or inhibitors in CCD841 CoN cells was performed using Lipofectamine RNAiMAX (ThermoFisher Scientific, Waltham, MA) (Li and Sarna, 2009). To assess the effect of miR-29b on gene expression, miR-29b mimics were transfected into H9c2 cells and the cells were harvested for RNA isolation 48 h later. To demonstrate that CCD841 CoN cells-secreted miR-29b suppresses BDNF in H9c2 cells, CCD841 CoN cells were transfected with miR-29b inhibitors or negative control miRNAs. Twenty-4 h later, the cells were treated with 10 ng/ml of human recombinant IL-1β for 24 h and the medium was removed. Next, the cells were incubated with fresh medium without IL-1β for 24 h and the medium was transferred to H9c2 cells. Twenty-4 h later, the H9c2 cells were harvested for RNA and protein extraction. To show that engineered exosomes rich in miR-29b dysregulate proteins such as BDNF in H9c2 cells, exosomes were isolated from the culture medium of CCD841 CoN cells via ultra-centrifugation and transfected with rno-miR-29b-3p mimics or negative control miRNA using ExoFectin sRNA-into-Exosome Kit (Cat. #P401, 101Bio, Mountain View, CA), followed by purification, resuspension in PBS, and incubation with H9c2 cells for 24 h. Total proteins were extracted for Western blot analysis of selected proteins.

### Animals and Procedures

Total ten 6-week old wild type C57BL/6J mice (n = 5 per group) were purchased from Envigo (Indianapolis, IN) and used in the preclinical studies. The mice were housed in the UTMB animal facility with 12 h light/12 h dark cycle, a temperature range of 24–26°C, and a relative humidity of 40–60%. Regular chow and water were provided *ad libitum*.

To determine if miR-29b packaged in plasma exosomes affects gene expression in the adult heart to trigger molecular remodeling, we isolated plasma exosomes from wild type C57BL/6J mice, transfected the exosomes with mmu-miR-29b-3p mimics or negative control miRNAs, and purified the exosomes. The engineered exosomes were then re-suspended in PBS and administered to live mice by tail vein injection under anesthesia with isoflurane (4% for induction and 1% for maintenance) (Li et al., 2009). Twenty-4 h later, the mice were decapitated under deep plane of anesthesia and individual hearts were collected, snap-frozen in liquid nitrogen, and pulverized for total protein extraction. This study was carried out in strict accordance with the recommendations in the Guide for the Care and Use of Laboratory Animals of the National Institutes of Health. All procedures were approved by the Institutional Animal Care and Use Committee, The University of Texas Medical Branch at Galveston (Protocol # 1512071A).

### Patient Information and Sample Collection

Human studies were approved by the Institutional Ethics Committee of Binzhou Medical University Hospital. Six patients (2 males and 4 females) with newly diagnosed UC and 4 healthy control subjects with no malignancy (2 males and 2 females) were enrolled in this study at Binzhou Medical University Hospital from September 2018 to May 2019. The personal information of all patients involved in this study was blinded to the investigators, and an IRB exemption was approved. The clinical and pathologic characteristics of the patients were summarized in online Supplementary Table S1. Approximately 20 ml of peripheral blood was collected from each patient and centrifuged (2000 x g, 4°C) for 10min, resulting in about 10 ml of plasma, which was aliquoted, snap-frozen in liquid nitrogen, and stored in a −80°C freezer before being shipped in dry ice for exosomal miRNA profiling.

### Exosome Isolation, Labeling, and RNA Extraction

The plasma was mixed with ExoQuick Exosome Precipitation Solution, and the exosomes were precipitated according to the manufacturer’s instructions (System Biosciences, Palo Alto, CA). Purified exosomes were labeled with the green fluorescent linker PKH67 (Sigma) as described previously (Yang et al., 2011). Total RNA was isolated using Trizol (Invitrogen, Waltham, MA). The quantity and integrity of RNA were assessed using the Qubit^®^2.0 (Life Technologies, Carlsbad, CA) and Agilent 2,200 TapeStation (Agilent Technologies, Santa Clara, CA) separately.

### Library Construction and Next-Generation Sequencing

Library preparation and small RNA sequencing was performed by RiboBio (Guangzhou, China). One microgram of total RNA of each sample was used to prepare small RNA libraries using NEBNext^®^ Multiplex Small RNA Library Prep Set for Illumina (NEB, Ipswich, MA) according to the manufacturer’s instruction. Total RNA samples were fractionated on a 15% Tris-borate-EDTA polyacrylamide gel (Invitrogen), and small RNAs ranging between 18 and 30 nucleotides were recovered and reverse transcribed, followed by PCR amplification. The libraries were sequenced on a HiSeq 2,500 platform (Illumina, San Diego, CA).

### Bioinformatics Analysis of Small RNA-Sequencing Data

To obtain clean reads, the raw reads were processed by filtering out the reads containing adapter or poly “N,” with low quality, or smaller than 17 nt using FASTQC. Mapping reads were obtained by mapping the clean reads to the reference human genome using BWA. miRDeep2 was used to identify known mature miRNAs based on miRBase21 (www.miRBase.org) and to predict novel miRNAs. Databases of Rfam12.1 (www.rfam.xfam.org) and pirnabank (www.pirnabank.ibab.ac.in) were used to identify rRNA, tRNA, snRNA, snoRNA and piRNA with BLAST. The miRNA expression levels were calculated using the RPM (Reads Per Million) values. Differential expression between two sets of samples was calculated using edgeR algorithm according to the criteria of (log2 (fold change)) ≥ 1 and *p*-value < 0.05. TargetScan, miRDB, miRTarBase, and miRWalk were used to predict target genes of selected miRNA. KOBAS was used to further GO and KEGG pathway analysis.

### RT-qPCR

Total RNA containing miRNA was extracted using the miRNeasy Mini Kit (QIAGEN, Valencia, CA), followed by cDNA synthesis using SuperScript III First-Strand Synthesis System or TaqMan™ Advanced miRNA cDNA Synthesis Kit (Invitrogen). The mRNA and miRNA levels were quantitated using TaqMan-based qPCR, with *Gapdh* and *18S rRNA* as internal controls, respectively (Zhong et al., 2018; Kline et al., 2019). All TaqMan^®^ Assays were purchased from Applied Biosystems (Waltham, MA).

### Western Blot

Western blotting was performed as described previously (Zhong et al., 2018; Kline et al., 2019). Primary antibodies were as follows: anti-BDNF rabbit polyclonal (Cat. # OSB00017W, 1:1,000) (ThermoFisher Scientific), anti-cleaved Caspase-3 rabbit polyclonal (Cat. # 9661S, 1:1,000), and anti-GADPH rabbit polyclonal (Cat. # 5174S, 1:1,000) (Cell Signaling, Danvers, MA). All blots were scanned using an Odyssey Infrared Imaging System (LI-COR Biosciences, Lincoln, Nebraska). Band density was determined using LI-COR Image Studio Software.

### Statistical Analysis

The comparisons of means among groups were analyzed by one way ANOVA, and the Dunn Multiple Comparison Test was further used to determine significant differences between groups. The association between circulating patients’ clinical–pathologic parameters was compared using Pearson χ^2^ test. All statistical analyses were performed using the SPSS package (version 13.0). A value of *p <* 0.05 was considered statistically significant. All authors had access to the study data and had reviewed and approved the final manuscript**.**

## Synopsis

Differentially expressed plasma exosomal miRNAs in patients with ulcerative colitis could be used for diagnosis and evaluation of therapeutic response. Exosomal miR-29b of gut origin might impair heart function by suppressing brain-derived neurotrophic factor in patients with ulcerative colitis.

## Data Availability

The datasets presented in this study can be found in online repositories. The names of the repository/repositories and accession number(s) can be found in the article/Supplementary Material.

## Abbreviations

IBD, inflammatory bowel disease; UC, ulcerative colitis; BDNF, brain-derived neurotrophic factor; CD, Crohn’s disease; TNFα, tumor necrosis factor alpha; IL-1β, interleukin-1 beta; PCA, principal component analysis; KEGG, Kyoto encyclopedia of genes and genomes; GO, gene ontology; TrkB, tropomyosin-related kinase receptor B; IBS, irritable bowel syndrome; RT-qPCR, reverse transcriptase-quantitative polymerase chain reaction; GAPDH, glyceraldehyde 3-phosphate dehydrogenase; RPM, reads per million; BLAST, basic local alignment search tool; KOBAS, KEGG orthology based annotation system.
