# Supplementary material for: Exosomal miR-29b of Gut Origin in Patients With Ulcerative Colitis Suppresses Heart Brain-Derived Neurotrophic Factor
Source: Front Mol Biosci. 2022 Feb 22;9:759689. doi: 10.3389/fmolb.2022.759689 (PMC8902158; doi:10.3389/fmolb.2022.759689)
Supplement: Supplementary file 2 [file Presentation1.pdf]

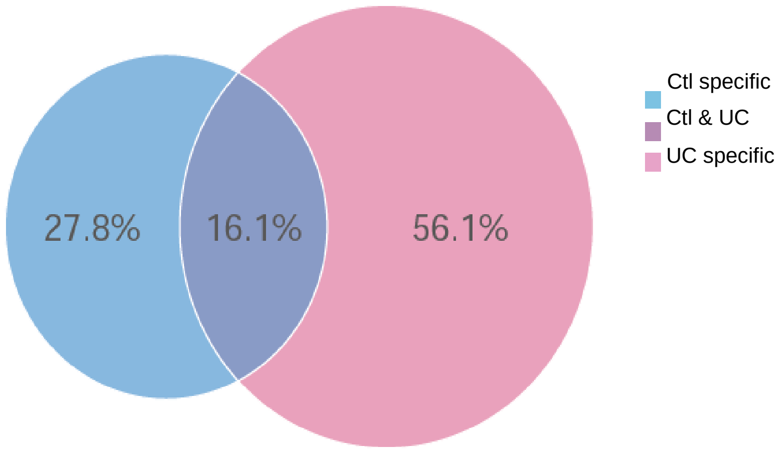

Fig S1. Venn chart showing unique and common miRNA sequences of UC patients and control subjects.

Chr20_10902

Provisional ID	:	chr20_10902
Score total	:	9903.8
Score for star read(s)	:	3.9
Score for read counts	:	9896.3
Score for mfe	:	2
Score for randfold	:	1.6
Score for cons. seed	:	
Total read count	:	19423
Mature read count	:	19071
Loop read count	:	0
Star read count	:	352

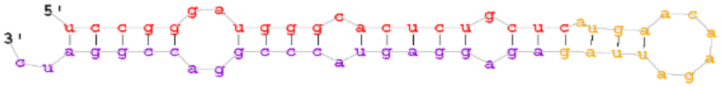

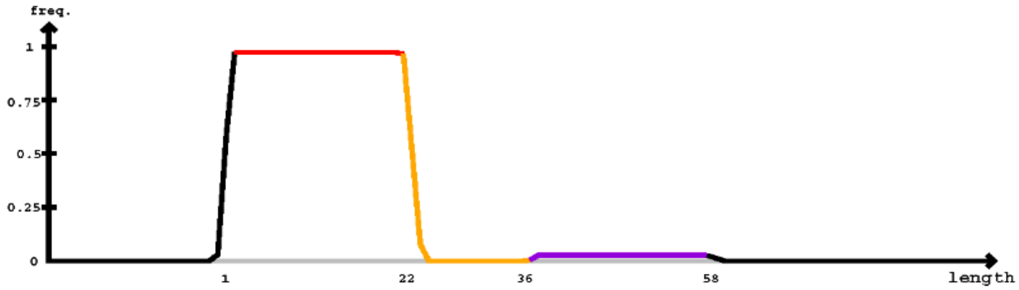

Mature Star

5'- cccugggcgggggcacaggcuccgggaugggcacucugcucaugaacaagauuagagaggaguacccggaccggaucaugaauuccuucagcgucaugccuucucccaag -3' obs

cccugggcgggggcacaggcuccgggaugggcacucugcucaugaacaagauuagagaggaguacccggaccggaucaugaauuccuucagcgucaugccuucucccaag exp

Fig S2. The nucleic acid sequence of miR-10902, a newly predicted miRNA.

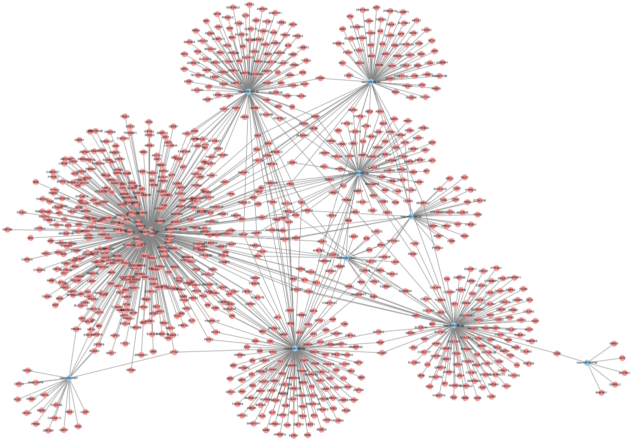

Fig S3. Regulatory network of 13 miRNAs upregulated in patients with UC.

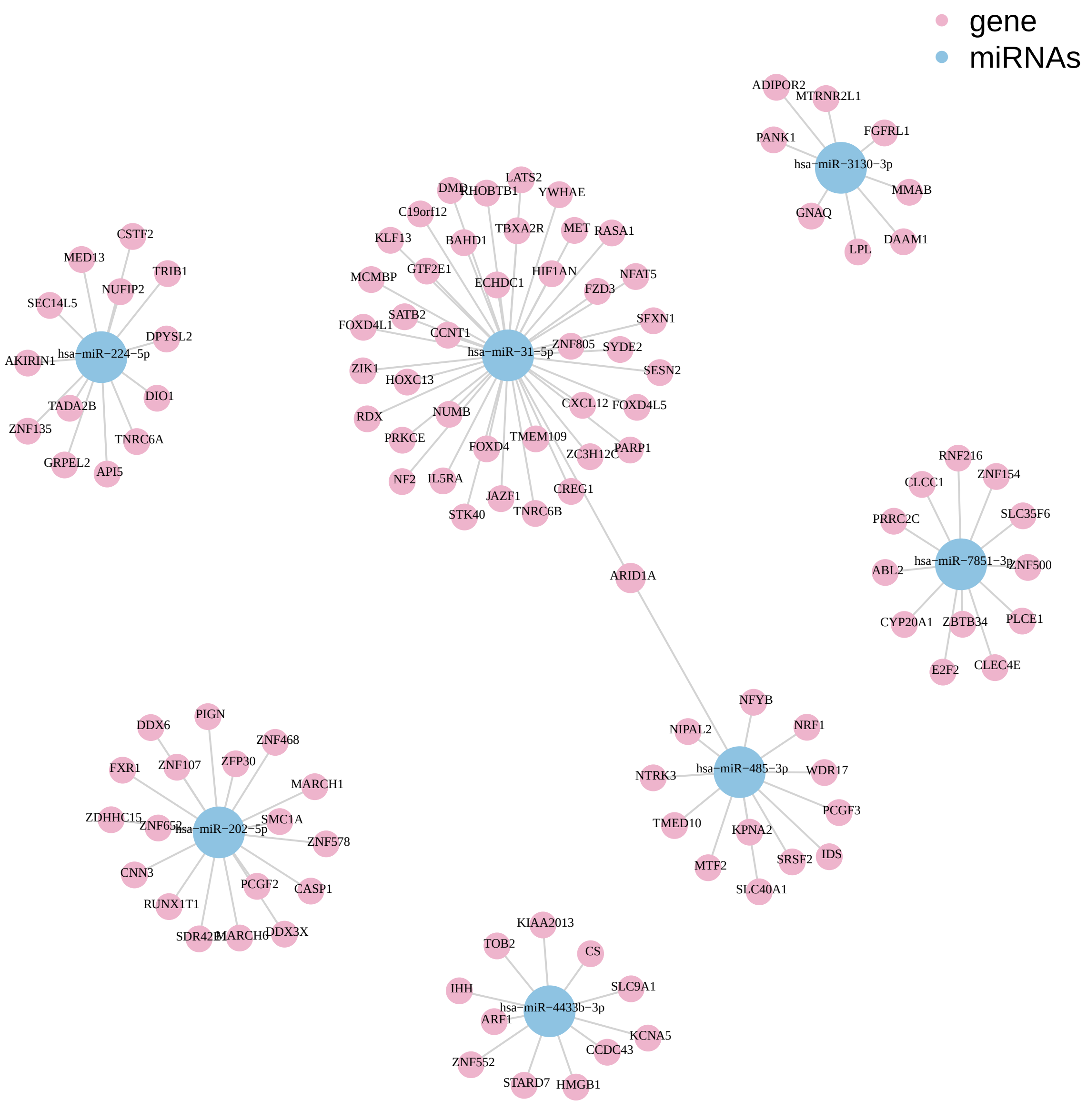

Fig S4. Regulatory network of 7 miRNAs downregulated in patients with UC.

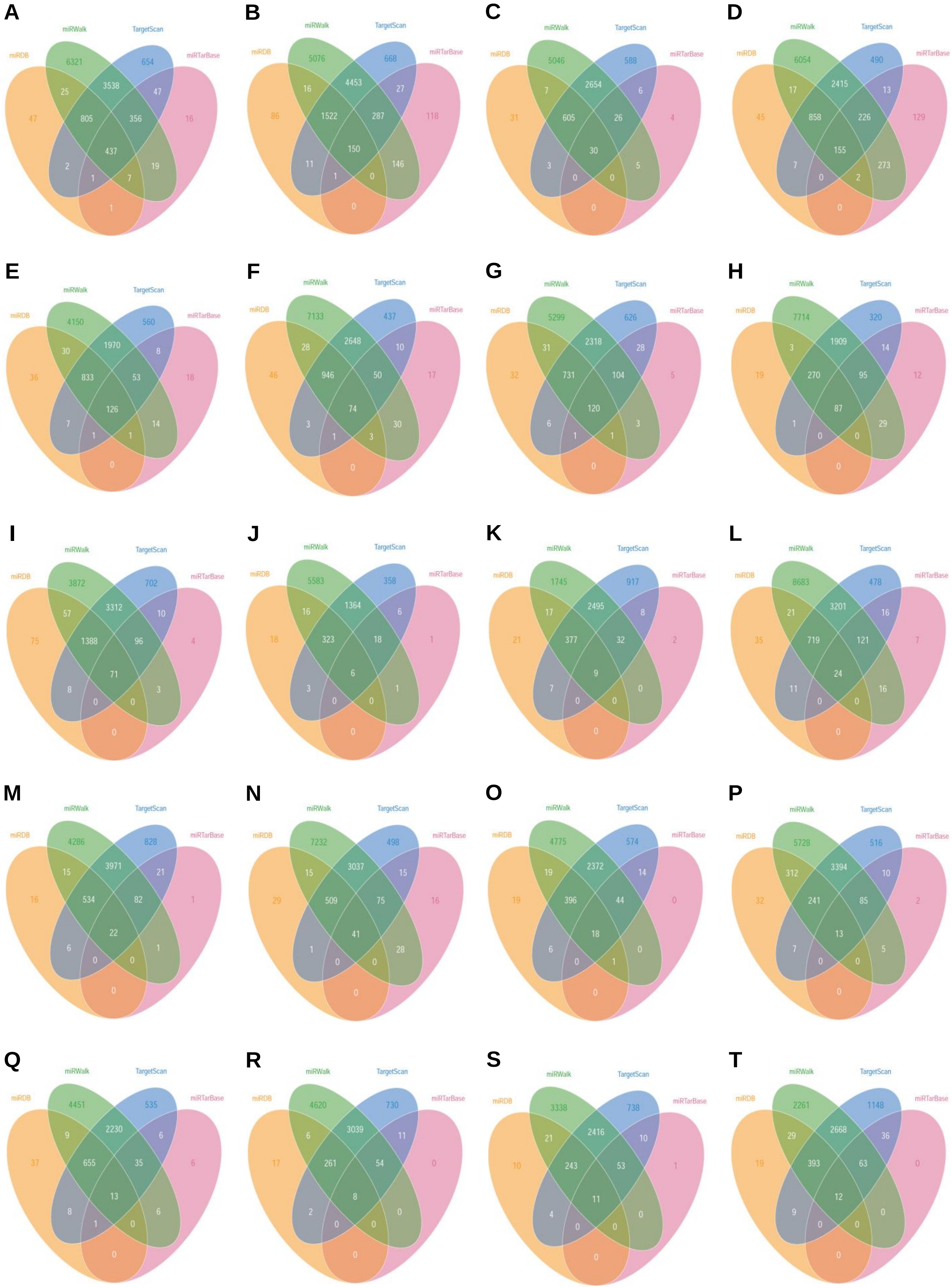

Fig S5. Venn diagrams showing the target gene numbers of each differentially expressed miRNA. Target genes were predicted by using miRDB, miRWalk, TargetScan, and miRTarBase software. **A.** hsa-miR-20b-5p; **B.** hsa-miR-186-5p； **C.** hsa-miR-194-5p； **D.** hsa-miR-218-5p； **E.** hsa-miR-29b-3p； **F.** hsa-miR-96-5p; **G.** hsa-miR-363-3p; **H.** hsa-miR-503-5p; **I.** hsa-miR-548au-5p; **J.** hsa-miR-624-5p; **K.** hsa-miR-942-3p; **L.** hsa-miR-1303; **M.** hsa-miR-4487; **N.** hsa-miR-31-5p; **O.** hsa-miR-202-5p; **P.** hsa-miR-224-5p; **Q.** hsa-miR-485-3p; **R.** hsa-miR-3130-3p; **S.** hsa-miR-4433b-3p; **T.** hsa-miR-7851-3p.

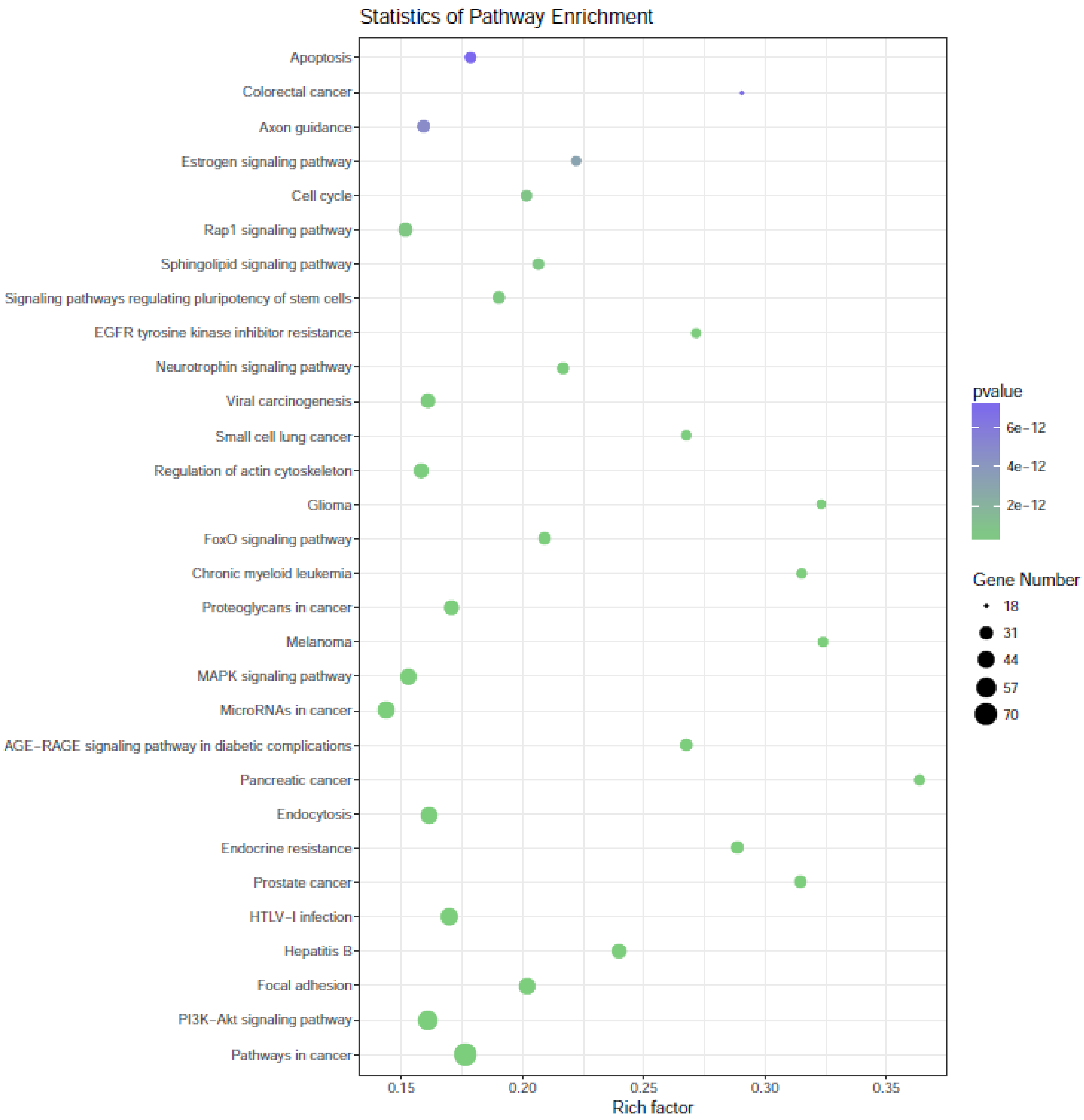

Fig S6. The bubble map of KEGG pathway enrichment analysis of the upregulated miRNAs in UC patients. The X axis shows rich factors representing enrichment degrees. The Y axis shows the names of the enriched pathways. The area of each node represents the number of enriched host genes corresponding to the upregulated miRNAs. The P value is represented by a color scale, high significance in purple and low significance in green.

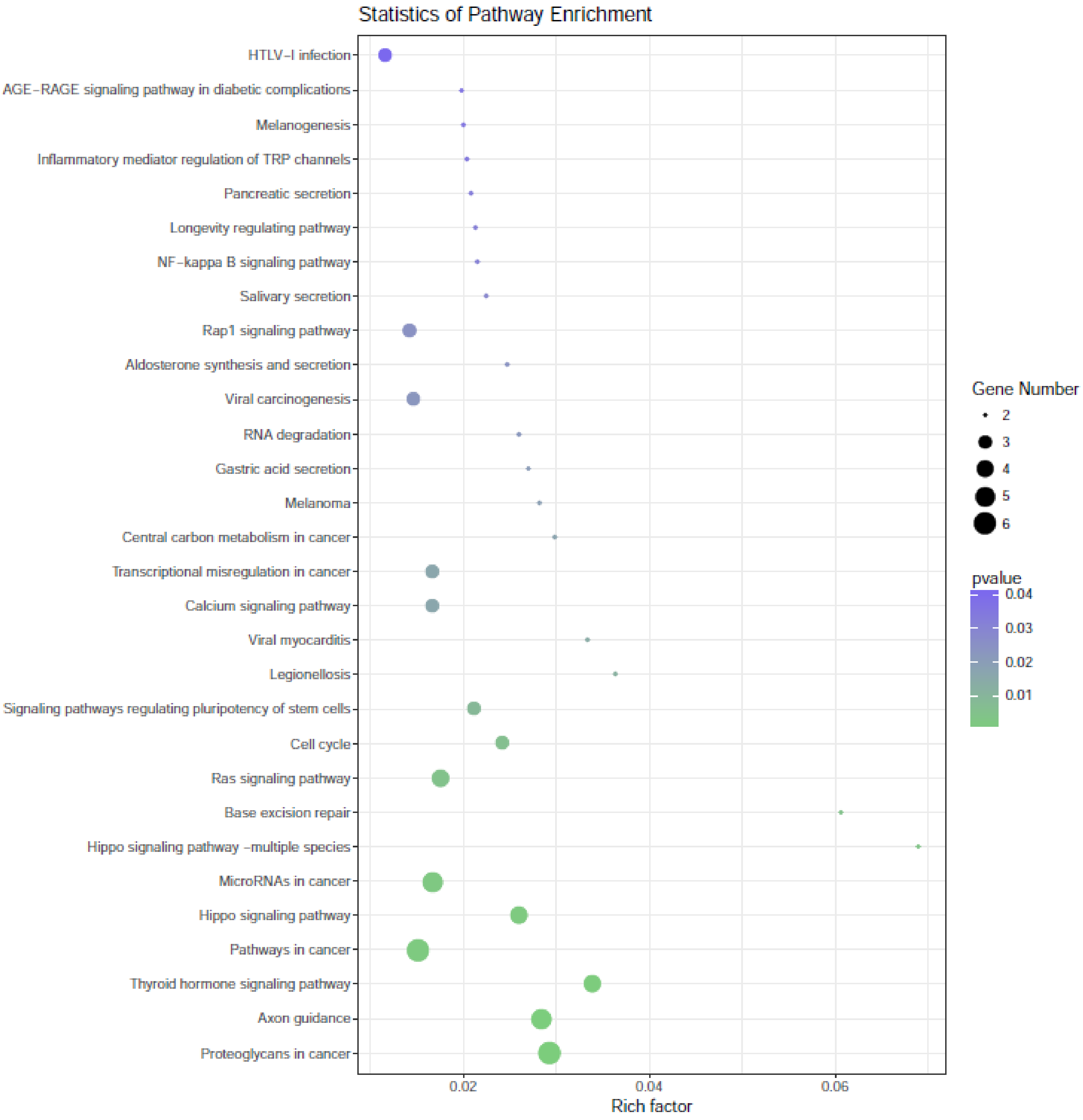

Fig S7. The bubble map of KEGG pathway enrichment analysis of the downregulated miRNAs in UC patients. The X axis shows rich factors representing enrichment degrees. The Y axis shows the names of the enriched pathways. The area of each node represents the number of enriched host genes corresponding to the downregulated miRNAs. The P value is represented by a color scale, high significance in purple and low significance in green.

Table S1. Clinical-pathologic characteristics of 6 patients with UC

Clinical-pathological variables	No. of patients
Gender	
Female	4
Male	2
Age	
≤40	2
>40	4
Montreal typing	
E1	1
E2	1
E3	4
Endoscopic Mayo scores	
1	0
2	4
3	2

Table S2. The top 6 newly predicted miRNAs

Chromosome location	Mature miRNA sequence	Total score	Reads count
chr20_10902	uccgggaugggcacucugcuc	9903.8	19423
chr17_7016	cuuccugguuccggacagcugg	247.6	491
chrUn_gl000220_22024	ccgccgcggcggccgucgggu	182.1	352
chr4_15656	uucguggugagucuuaacagcu	141.4	283
chr1_8924	ucaggccaggcugggaggaug	126.9	245
chr2_13204	cucuccgccaccuccaccgcgg	109.9	212

Novel miRNAs were predicted using miRDeep2.
